# A Conversation with Sambuddha Misra

**DOI:** 10.1021/acscentsci.5c00557

**Published:** 2025-04-09

**Authors:** Payal Dhar

Fifty percent of the photosynthesis happens in the ocean. To sustain all that life, oceans need rivers to supply nutrient-rich sediments and dissolved minerals. Sambuddha Misra, a chemical oceanographer at the Indian Institute of Science in Bengaluru, researches how these materials get transported—from the glacier-melt stage to when they flow to the sea—and how that movement of nutrients helps maintain ecosystems. Understanding this process, he says, gives you an idea of what keeps the planet habitable.

**Figure:**
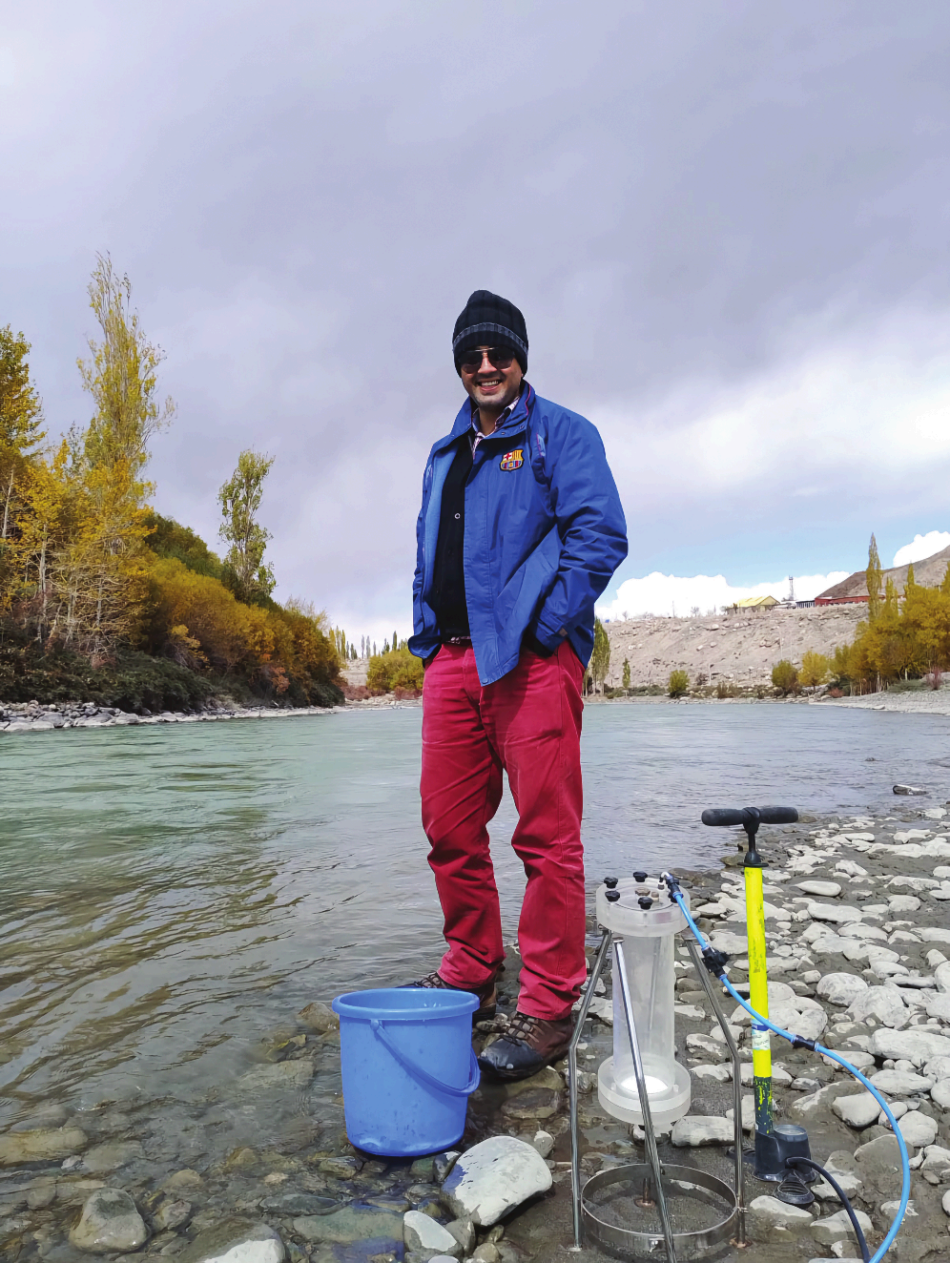
Sambuddha Misra on the banks of the Indus river, collecting river water samples for trace metal analysis. Credit: Parul Ashodiya and Kribina Pathak.

Misra’s lab analyzes isotopes of lithium, boron, magnesium, potassium, and lead trapped in marine carbonates. He and his colleagues use these data to reconstruct chemical shifts that happened in the environment decades or even millennia ago. These include historic changes in atmospheric carbon dioxide concentrations, the transport of metals in large river systems, and the role of reverse weathering—when dissolved components like aluminum, iron, manganese, and silica come together to form clay particles—in modulating seawater chemistry.

In a paper published late last year, Misra and colleagues pieced together the history of lead pollution in the Indian Ocean between 1989 and 2013 by measuring the chemical content of coral in reefs of Lakshadweep, an archipelago off of India’s southwestern coast. They found that in this quarter-century period, lead concentrations in the western Indian Ocean had doubled, and they concluded that most of the lead came from anthropogenic sources such as aerosols from the surrounding areas.

Payal Dhar spoke with Misra about changes in water chemistry and lead concentration in ocean water and how these changes affect people and the environment. This interview was edited for length and clarity.

## How do you reconstruct historical changes in sea chemistry?

It is quite intriguing. We use foraminifera and corals for this. Foraminifera [tiny, single-celled organisms] have a life cycle of about a month in the ocean; then they die and settle, becoming part of the sediment. So if you take a core from the ocean floor and date it using radiocarbon or uranium–thorium [dating] or oxygen isotope stratigraphy, you have an archive that averages seawater chemistry through time.

Corals [used for the 2024 study] have an annual growth band, which can be dated using radiocarbon. If you analyze the chemistry, it helps reconstruct past changes in the seawater. We use proxies for that—a good example being boron isotopes for pH.

Setting up lead isotope [analysis] was very difficult. Lead is ubiquitous; every particle of dust carries lead with it. So you need a dust-free lab. Then, from corals you are extracting less than a nanogram of lead, and you have to measure the isotope ratio on that subnanogram quantity with mass spectrometry. Keep in mind that the lead has to be purified by ion-exchange chromatography. This makes the entire exercise fiendishly difficult.

## You concluded that lead concentration in the western Indian Ocean doubled over 24 years. What caused that massive increase?

The lead primarily comes from anthropogenic activity. There have been a lot of industrial sources of lead over the past 20 years, with rapid industrialization in India and other sub-Saharan African countries.

No river can carry lead to a remote location like Lakshadweep. So if you observe an increase in lead concentrations there, you know that there is a lot of lead in the dust carried by the wind. And where does the lead dust come from? Industrial lead emission, mostly through coal burning.

The surface environment across India is very badly contaminated with lead. Coal and every industry that uses coal are major polluters. Until 2000, we [in India] had been emitting lead through gasoline. Without industrial emission, we should have observed either a plateau or a decrease in lead concentrations.

In addition, what I would call a ticking time bomb is the recycling of lead-acid batteries. Most of it is done in a makeshift manner, not following any protocols.

## How does that lead affect people and the environment?

Lead has a huge impact on humans. A study in the US looked into lead concentration in the blood and its association with violent crimes. Lead is also known to cause neurological problems, arthritis, [and possibly cancer]. It is a poison our body cannot get rid of.

If you have increased heavy-metal concentration in the ocean, then photosynthesis will be hampered. For instance, cadmium is needed for photosynthesis, but excess cadmium is toxic. Same with lead if concentrations go beyond a certain value. We really do not understand how well lead is taken up by organisms and if it is bioaccumulative in nature with every passing trophic level.

## How does changing river chemistry affect lead concentrations?

Lead is not very water-soluble, so when we look at river water, we do not find a lot of lead in it. But when we look at river sediments, we find an enormous amount of lead embedded in them. When river water becomes acidic, like from industrial effluent, all the metal bound to the sediment is released.

Dissolved oxygen can also affect lead concentrations. Whenever you have a system deprived of dissolved oxygen, then many of these metals will become soluble because most metals are soluble in a reduced form rather than an oxidized form. A classical example is iron, which can be in a +2 or +3 oxidation state. Iron(II) is water-soluble; iron(III) is not.

## What can or should be done about the lead levels in the Indian Ocean?

Once it hits the natural system—given the widespread contamination of lead—there is very little we can do. But what we can definitely do is stop the emission. We need to sequester the lead at the source, because once it gets into the environment, all bets are off.

## Payal Dhar is a freelance contributor to

Chemical & Engineering News, *an independent news publication of the American Chemical Society.*

